# Complete Right Ventricular Thrombus in Pediatric Ebstein Anomaly

**DOI:** 10.1016/j.case.2026.02.009

**Published:** 2026-04-27

**Authors:** Mamta Shah, Shreya Sreeram, Jesse Lee, Rod Shinozaki

**Affiliations:** a Division of Pediatric Critical Care, Department of Pediatrics, Loma Linda University Children’s Hospital, Loma Linda, California; b Department of Pediatrics, Loma Linda University Children’s Hospital, Loma Linda, California; c Division of Pediatric Cardiology, Department of Pediatrics, Loma Linda University Children’s Hospital, Loma Linda, California

**Keywords:** Ebstein anomaly, Starne’s procedure, Intracardiac thrombus, Anticoagulation, Single-ventricle physiology

## Abstract

• Complete RV thrombus was observed in Ebstein anomaly while on aspirin therapy.
• RV thrombus can result in myocardial infarction and lethal LV compression.
• Standardized anticoagulation is limited in fenestrated RV exclusion physiology cases.

## Graphical abstract

**Figure:**
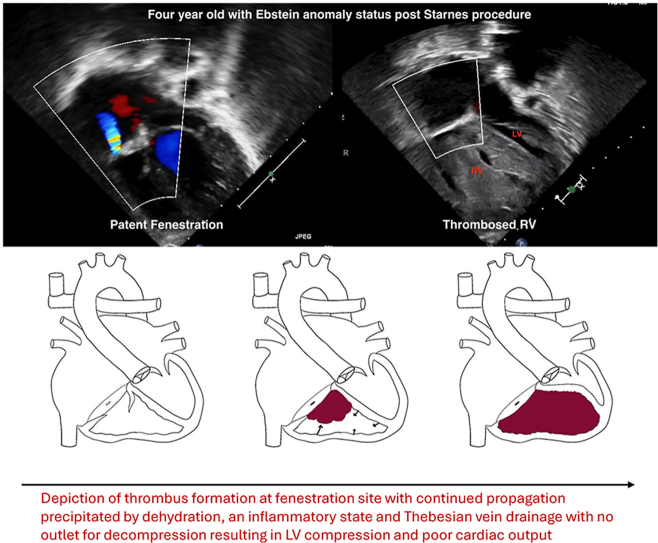

## Introduction

Ebstein anomaly is a rare cardiac anomaly accounting for 0.3% to 0.5% of congenital heart defects. The anatomic defect resulting in Ebstein anomaly involves inferior displacement of the tricuspid valve into the right ventricle, resulting in tricuspid regurgitation, atrialization of the right ventricle, and right ventricular (RV) hypoplasia. Disease severity can vary significantly, with severe forms requiring surgical intervention.^1^

The case presented describes the complications of an RV thrombus in a patient with Ebstein anomaly with Glenn physiology. The case highlights the pathophysiologic findings of tamponade physiology and explores current evidence for both prevention and treatment of thrombi in single ventricle patients.

## Case Presentation

A 4-year-old boy with Ebstein anomaly presented to the emergency department with 2 days of increased work of breathing and dehydration secondary to human metapneumovirus. Surgical history was notable for a fenestrated RV exclusion (Starnes) procedure with a Blalock-Taussig-Thomas shunt, a subsequent Glenn shunt, and failed Fontan palliation secondary to pulmonary hypertension with intraoperative take-down of Fontan.

**Figure 1:**
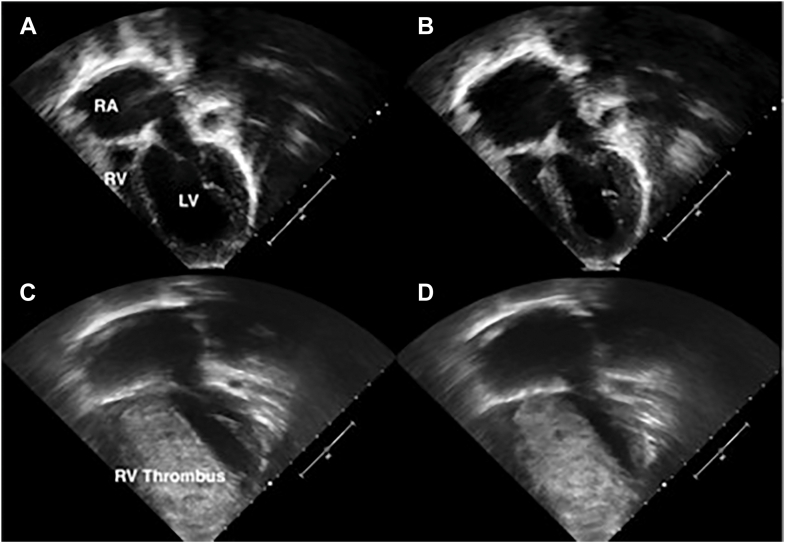
Two-dimensional transthoracic echocardiography, apical four-chamber diastolic **(A, C)** and systolic **(B, D)** views, demonstrates baseline findings with a normal postoperative right ventricle (RV) without thrombus (4 months prior; **A, B**) compared with current presentation with complete right ventricular thrombus and left ventricular compression **(C, D)**. *LV*, Left ventricle; *RA*, right atrium.

**Figure 2:**
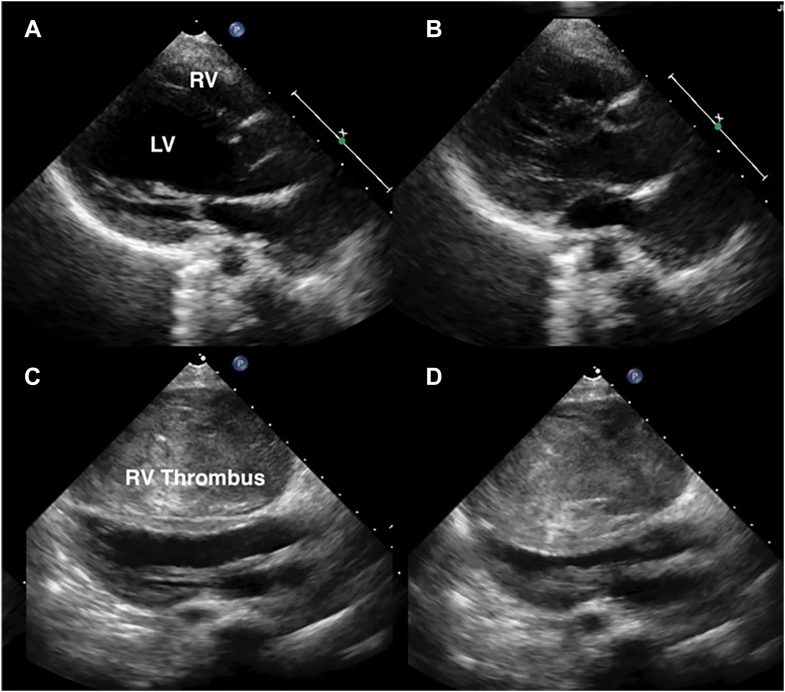
Two-dimensional transthoracic echocardiography, parasternal long-axis diastolic **(A, C)** and systolic **(B, D)** views, demonstrates baseline findings with normal postoperative left ventricular size and septal position without right ventricular thrombus (4 months prior; **A, B**) compared with current presentation with complete right ventricular thrombus, left ventricular compression, and septal convexity **(C, D)**. *LV*, Left ventricle; *RV*, right ventricle.

**Figure 3:**
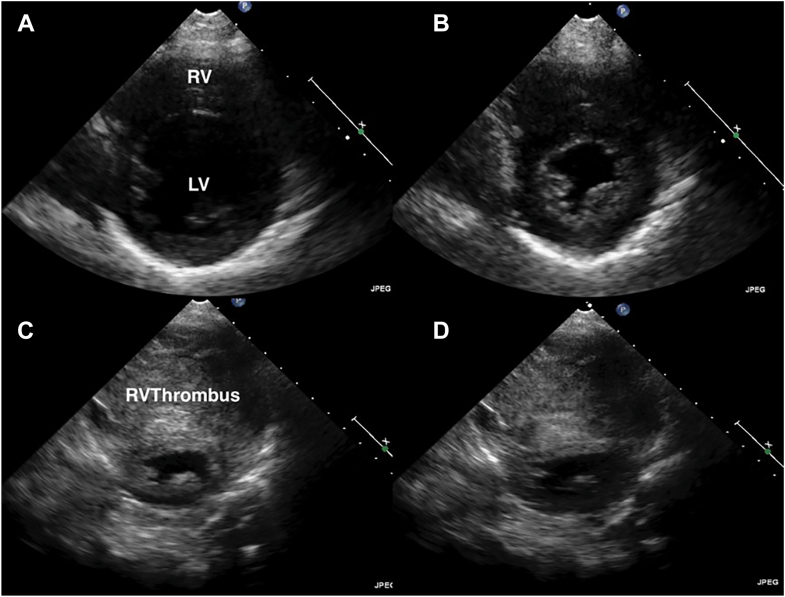
Two-dimensional transthoracic echocardiography, parasternal short-axis diastolic **(A, C)** and systolic **(B, D)** views, demonstrates baseline findings with normal postoperative left ventricular size and septal position without right ventricular thrombus (4 months prior; **A, B**) compared with current presentation with complete right ventricular thrombus, left ventricular compression, and septal flattening **(C, D)**. *LV*, Left ventricle; *RV*, right ventricle.

Echocardiography on admission was notable for the formation of a large RV thrombus without fenestration flow and left ventricular compression (Figure 1, Figure 2, Figure 3, Videos 1-3). As an outpatient, the patient was on daily aspirin with reportable compliance.

The case was discussed with both surgery and interventional cardiology; however, because of complex surgical history and poor wound healing, the team elected to pursue medical management and initiated a heparin infusion with plans for cardiac transplantation evaluation.

**Figure 4:**
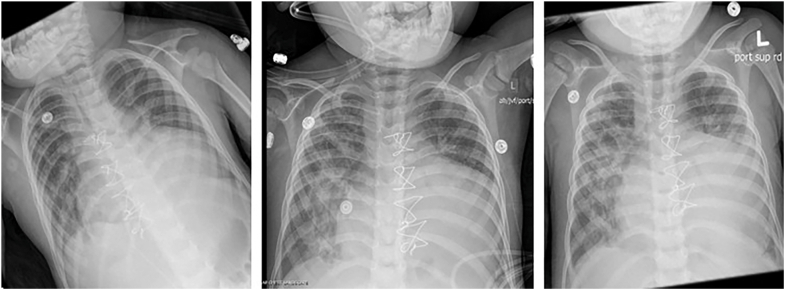
Findings on chest radiography from days 2 to 4 of admission demonstrate progressive worsening of bilateral opacities contributing to elevated pulmonary pressures and pulmonary hypertensive crisis.

**Figure 5:**
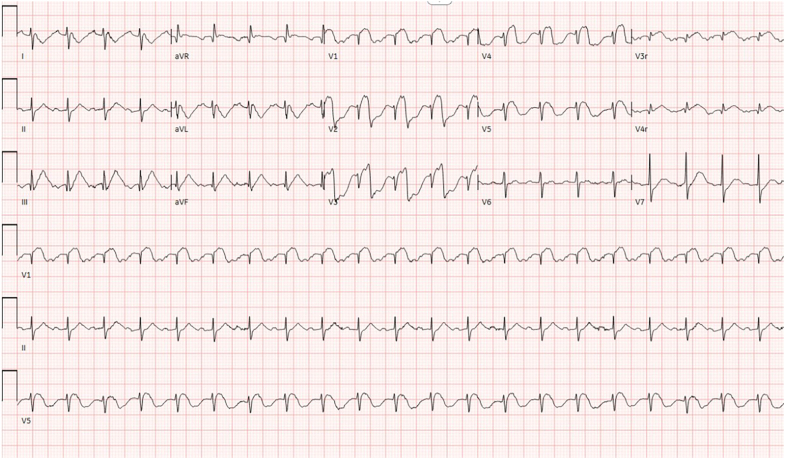
Twelve-lead electrocardiography demonstrates sinus tachycardia, first-degree atrioventricular block, and marked ST-segment elevation in leads V_1_ to V_5_, with reciprocal ST-segment depression in lead V_7_, consistent with an acute myocardial infarction.

On day 3 of admission, the patient began to experience respiratory distress requiring transition to noninvasive positive pressure ventilation. Radiologic imaging at that time demonstrated worsening pulmonary infiltrates (Figure 4). In the process of transitioning respiratory support, the patient became agitated and uncomfortable and experienced an acute pulmonary hypertensive crisis. Telemetry and 12-lead electrocardiography at that time demonstrated new ST-segment elevations (Figure 5), with an elevated troponin (>10,000 ng/L) concerning for myocardial infarction. Importantly, there was no preexisting coronary anomaly.

Surgical intervention was revisited, but with complex vasculature secondary to significant collateral vessels and a history of postoperative infections with an ongoing viral illness, medical management was continued and systemic tissue plasminogen activator (tPA: 0.05 mg/kg/h) was initiated for 12 hours. In that time, the patient’s hemodynamics substantially worsened, with decreasing mean arterial pressure as well as down-trending mixed venous saturations to 35%, compared with a baseline of 65%. Unfortunately, the patient developed worsening respiratory distress and during preparation for intubation experienced cardiac arrest without return of spontaneous circulation. Postmortem findings were unavailable, as the family declined to authorize an autopsy.

## Discussion

Ebstein anomaly is a congenital heart disease characterized by failure of delamination of the septal leaflet of the tricuspid valve, resulting in a variable degree of tricuspid regurgitation and RV size and function.^2^ Patients with severe forms of Ebstein anomaly undergo the Starnes procedure in infancy. During this procedure, the right ventricle is excluded from the pulmonary circulation, and pulmonary blood flow is supplied through a central shunt or a Blalock-Taussig-Thomas shunt.^2^ Patients subsequently follow the single-ventricle pathway, with transition to Glenn and then Fontan physiology.^3^ Given continued drainage of the Thebesian veins into the right ventricle, decompression through a fenestration and initiation of anticoagulation are warranted to decrease the risk for intracardiac thrombus (ICT) formation.

Although the right ventricle no longer serves a functional role after completion of the Starnes procedure, thrombus formation can substantially affect cardiac output and contribute to multiple complications.

A clot at the site of the fenestration most likely prevented adequate decompression of the right ventricle in our patient. The resultant stasis of blood in the setting of continued flow from the Thebesian venous drainage provided a nidus for thrombus propagation. The inflammatory cascade precipitated by the presence of a thrombus also contributed to a hypercoagulable state, increasing the risk for thromboembolic events, a particular concern in single-ventricle physiology.^4^

In this patient, RV thrombus formation caused a shift of the septum and subsequent compression of the left ventricle, resulting in pulmonary congestion and reduced cardiac output.

In addition, the occlusion of the fenestration and RV thrombus likely contributed to myocardial ischemia by increasing RV end-diastolic pressure and reducing coronary perfusion pressures, ultimately resulting in myocardial ischemia and infarction. Ventricular interdependence was thus overall compromised, leading to an overall reduced cardiac output.

This case details the extremely rare event of a complete RV thrombus formation. Although the risk for developing a thrombus is a known complication of Glenn physiology, the incidence is low. In a retrospective study assessing outcomes among the original cohort of 89 patients who underwent the Glenn procedure, none of the patients in the cohort had developed Glenn shunt thrombi over a 50-year period.^5^ We identified a report by Imanaka *et al*.^6^ describing two cases in which patients with pulmonary atresia and intact septum developed a RV thrombus in the immediate postoperative period after the Glenn procedure. Additionally, there was one report of a patient with Ebstein anomaly, status post fenestrated Fontan, who presented with an RV thrombus causing compression of the left ventricle. The patient underwent surgical excision of the thrombus, though survival outcomes were not detailed.^7^

We were unable to identify any reports of patients with Ebstein anomaly and Glenn physiology developing RV thrombus as in our case. Given the low incidence of thrombus formation after bidirectional cavopulmonary anastomosis, there is little consensus with regard to the appropriate regimen for anticoagulation prophylaxis in this patient population.

A review by Agarwal *et al*.^8^ analyzed 13 studies assessing outcomes in patients receiving thromboprophylaxis after the Glenn procedure. The review revealed significant variation in thromboprophylaxis strategies across studies with regard to single- vs dual-agent therapy, the selection of anticoagulant therapy, and the timing of treatment initiation. Current American Heart Association guidelines report that the use of antiplatelet therapy is reasonable in patients with superior cavopulmonary anastomosis and recommend the addition of antithrombotic therapy in high-risk patients. However, the recommendation is based on an expert consensus rather than supported by high-quality clinical trials.^9^

Overall, the prevalence of ICT in pediatrics is low; however, the associated morbidity and mortality are high. A retrospective study characterizing outcomes in pediatric patients with ICT reported a 39% mortality rate, with embolic events occurring in 13% of patients.^10^ Traditionally, transthoracic and transesophageal echocardiography have been the diagnostic tools for the identification of ICT.^11^ However, there has been a recent shift toward the use of cardiovascular magnetic resonance (CMR) given evidence of increased sensitivity in adult trials. A study by Curtis *et al*.^12^ evaluated echocardiographic and CMR findings among 119 patients with Fontan circulation and histories of thromboembolic event and showed that only 0.5% of patients were noted to have ICT on CMR. Such findings suggest that current diagnostic methods have overestimated the incidence of ICT, and a shift toward a different diagnostic modality may be warranted.^12^

With regard to treatment, the adult literature has pointed to the efficacy of catheterization-based interventions, but the data in pediatrics is limited, particularly in high-risk populations with congenital heart disease. Anticoagulation is currently the first-line therapy, with thrombectomy as warranted. However, there is limited evidence with regard to the duration of therapy, indications for prolonging therapy, or the impact of thrombus size on treatment.^13^ The role of systemic tPA for anticoagulation remains unclear, though a recent study by Olgun *et al*.^14^ demonstrated complete lysis of ICT or arterial thrombi in 17 of 22 pediatric patients, thereby demonstrating the efficacy of tPA for patients similar to the one described earlier.

## Conclusion

This case highlights the potential complication in patients with RV exclusion procedures and significant ICT. Although our patient was not a surgical candidate and was treated with systemic tPA, this scenario raises important concerns about additional anticoagulation and echocardiographic monitoring during illness to prevent such events. Further research in medical management and prophylactic strategies is warranted.

## Ethics Statement

The authors declare that the work described has been carried out in accordance with The Code of Ethics of the World Medical Association (Declaration of Helsinki) for experiments involving humans.

## Consent Statement

Complete written informed consent was obtained from the patient (or appropriate parent, guardian, or power of attorney) for the publication of this study and accompanying images.

## Funding Statement

The authors declare that this report did not receive any specific grant from funding agencies in the public, commercial, or not-for-profit sectors.

## Acknowledgments

We thank the sonographers within the Division of Pediatric Cardiology at Loma Linda University Children’s Hospital for the acquisition of images related to this case.

## Disclosure Statement

The authors report no conflicts of interest.

## Supplementary data

Video 1 Two-dimensional transthoracic echocardiography, apical four-chamber view with color flow Doppler obtained 4 months prior *(left)* with normal left ventricular size and function, no RV thrombus, and patent fenestration flow *(blue)* compared with current presentation *(right)* with small (underfilled) left ventricular size, normal left ventricular function, dilated right ventricle with complete intracavitary RV thrombus, absent fenestration flow, and left ventricular compression.

Video 2 Two-dimensional transthoracic echocardiography, parasternal long-axis view obtained 4 months prior *(left)* with normal left ventricular size and function, normal interventricular septal position, and no RV thrombus compared with current presentation *(right)* with small (underfilled) left ventricular size, normal left ventricular function, dilated right ventricle with complete intracavitary RV thrombus, abnormal (leftward bowing) interventricular septal position, and left ventricular compression.

Video 3 Two-dimensional transthoracic echocardiography, parasternal short-axis view obtained 4 months prior *(left)* with normal left ventricular size and function, normal interventricular septal positioning, and no RV thrombus compared with current presentation *(right)* with small (underfilled) left ventricular size, normal left ventricular function, dilated right ventricle with complete intracavitary RV thrombus, and abnormal (leftward bowing and flattening) of the interventricular septum with left ventricular compression.
